# Enhanced methane production with co-feeding spent coffee grounds using spare capacity of existing anaerobic food waste digesters

**DOI:** 10.1038/s41598-024-54610-y

**Published:** 2024-02-23

**Authors:** Danbee Kim, Junho Cha, Changsoo Lee

**Affiliations:** 1 Department of Urban and Environmental Engineering, Ulsan National Institute of Science and Technology (UNIST), 50 UNIST-Gil, Eonyang-Eup, Ulju-Gun, Ulsan, 44919 Republic of Korea; 2 Graduate School of Carbon Neutrality, Ulsan National Institute of Science and Technology (UNIST), 50 UNIST-Gil, Eonyang-Eup, Ulju-Gun, Ulsan, 44919 Republic of Korea; 3 Gwangju Clean Energy Research Center, Korea Institute of Energy Research, 25, Samso-Ro 270Beon-Gil, Buk-Gu, Gwangju, 61003 Republic of Korea

**Keywords:** Environmental biotechnology, Environmental microbiology

## Abstract

With increasing coffee consumption worldwide, the efficient and sustainable management of spent coffee grounds (SCG) has become increasingly challenging. This study investigated the anaerobic co-digestion of small amounts of SCG with food waste (FW) at increasing co-feeding ratios of 1:100–1:10 (volatile solids basis) to assess the possibility of SCG treatment using the spare capacity of existing anaerobic digesters. Co-feeding SCG increased methane production compared to FW mono-digestion in the tested range of co-feeding ratios without compromising process stability. Methane yield did not further increase when the SCG/FW ratio increased above 4%, and process failure occurred at a 1:10 co-feeding ratio without trace element supplementation. The enhanced methanogenic performance was attributed to increased protein removal efficiency, which was potentially related to the promotion of peptide hydrolysis. The overall results suggest that co-feeding appropriate small amounts of SCG to FW digesters can be a realistic sustainable option for SCG management.

## Introduction

World coffee consumption has continuously increased, and the global production of spent coffee grounds (SCG) is estimated at 18 million wet tonnes in 2021^1^. Annual SCG production in Korea increased approximately 1.6-fold, from 93,397 to 149,038 tonnes, between 2012 and 2019^2^. Although SCG have potential uses as a compost, construction material, and biofuel feedstock, most are disposed of by incineration or landfills, potentially creating an additional environmental burden^3^. Incineration of SCG with high moisture content is energy-intensive, and uncontrolled degradation of SCG in soil and water bodies can cause serious pollution^4^. Therefore, a more eco-friendly and sustainable way of managing SCG is in urgent need.

Methanation through anaerobic digestion (AD) has been considered an attractive approach for the treatment of organic-rich SCG because of its ability to convert organic carbon to biogas, a carbon–neutral energy carrier. AD is a well-established technology commonly used to treat high-strength organic wastes such as food waste (FW), livestock manure, and sewage sludge. Early studies in the 1980–1990s concluded that stable long-term AD of SCG as a sole substrate (i.e., mono-digestion) cannot be achieved, primarily because of the lack of nutrients and trace elements in SCG^5,6^. Another characteristic of SCG that makes its AD difficult is their high level of poorly bioavailable lignocellulosic materials^7,8^. Co-digestion of SCG with other organic wastes with complementary properties has been demonstrated as a viable strategy through which to solve these problems in many studies in continuous as well as batch cultures^7,9–13^. Co-digestion can improve the digestibility of the resulting substrate mixture by improving the balance of macro- and micronutrients, diluting inhibitory substances, and increasing buffering capacity^7^.

Previous SCG co-digestion studies in continuous cultures have mostly focused on the effects of co-substrates on digester performance and stability with SCG as the main substrate^9,11–13^. Qiao et al.^11^ report that an anaerobic continuously stirred tank reactor (CSTR) did not reach a steady state with a 9:1 mixture (dry weight basis) of SCG and waste-activated sludge but did with an 85:15 mixture under thermophilic conditions. Zhang et al.^13^ operated thermophilic CSTRs co-digesting SCG with waste-activated sludge at dry weight ratios of 7:3 and 8:2, and stable operation was achieved without any external supply of nutrients for both substrate mixtures. Kim et al.^12^ reported that, in a mesophilic CSTR, co-digesting SCG with macroalgal *Ulva* slurry (25% on a chemical oxygen demand (COD) basis) improved digester performance as compared to SCG mono-digestion. Given the relatively low biodegradability of SCG, it seems more practical to add small amounts of SCG as a co-substrate to anaerobic digesters handling other wastes produced in much larger amounts, such as FW and livestock manure. This approach is attractive, as it allows treating SCG using the spare capacity of existing anaerobic digesters, avoiding the need for additional facilities and enabling flexible SCG management. However, limited research has been conducted to explore this potential use, and more studies on the use of SCG as an additional substrate are needed.

In Korea, approximately 5.2 million tonnes of FW were produced in 2020^14^, which is more than 30 times larger than the SCG produced. A recent study tested the treatment of SCG as a co-substrate in mesophilic anaerobic CSTRs fed with FW as the base substrate^3^. Co-feeding SCG and FW at a ratio of 1:10 on a volatile solids (VS) basis resulted in severe process upset and performance deterioration as compared to FW mono-digestion, indicating that the mixing ratio of SCG should be lower to avoid compromising the methane potential of FW. Prabhudessai et al.^15^ reported that, in batch AD experiments with FW, small doses of caffeine (50–150 mg/L) stimulated methanation activity and resulted in significantly increased biogas production (up to > twofold) as compared to the no-caffeine control. This observation suggests that co-feeding SCG in small amounts may improve the performance of FW digesters, although the stimulation mechanism is unclear. The present study involved continuous anaerobic co-digestion of FW with small amounts of SCG (1–10% of FW on a VS basis) to investigate the possibility of treating SCG using the spare capacity of existing FW digesters without compromising the methanation of the substrate mixture. Duplicate digesters were monitored at various co-feeding ratios, with a focus on the effects of SCG on digester performance and microbial community dynamics.

## Materials and methods

### Reactor setup and operation

Duplicate anaerobic CSTRs were inoculated by filling the entire working volume (2 L) with anaerobic sludge from lab-scale FW digesters that had been continuously operated for over 38 months^16^. The inoculum sludge had a total solids (TS) content of 24.4 g/L, 68% of which was VS, and a total COD concentration of 26.7 g/L. The FW used as the base substrate was obtained from a canteen at UNIST on two occasions, primarily comprising rice, meat (mainly pork), and vegetables (mainly kimchi). The collected FW was finely ground into a slurry with a household blender and diluted to a VS concentration of 100 g/L (see Supplementary Table S1). The SCG used as the co-substrate were collected via a one-off sampling from a cafe at UNIST, dried under gentle conditions (55℃, ≥ 48 h), and desiccated at room temperature. Supplementary Table S1 shows the characteristics of the prepared base and co-substrates (i.e., FW and SCG). The reactors were initially fed only with FW (Phase 0) to collect the baseline performance data under FW mono-digestion conditions. Subsequently, during the following experimental phases (Phases 1–5), the reactors received SCG-FW mixtures at various mixing ratios of 1:100–1:10 on a VS basis (see Supplementary Table S2). This range was selected based on the observation that a 1:10 co-feeding ratio led to serious process instability in a previous study by the authors’ group^3^. Throughout the experiment, the reactors were operated at a constant hydraulic retention time (HRT) of 40 d, which was selected referring to previous co-digestion studies of SCG with FW^3^ and *Ulva* biomass^12^. Feeding was performed once a day, and the feed was amended with minimal amounts of key trace elements (100 mg Fe, 2 mg Co, and 1 mg Ni per liter)^16^ during Phases 0–4 to avoid the potential risk of process instability caused by their deficiency^3^. The reactors were run without pH control under mesophilic conditions (35 ± 2℃) throughout the experiment for 791 d.

### Analytical methods

Solids were measured according to the standard methods outlined by APHA-AWWA-WEF^17^. COD concentration was determined using a commercial kit (HS-COD-MR, HUMAS, Korea). Ammonium was measured by ion chromatography as previously described^3^. Biogas composition and volatile fatty acid (VFA) concentrations were analyzed by gas chromatography as previously described^18^. Samples for ion chromatography and soluble COD measurements were prepared by filtration through a membrane syringe filter (0.45-μm pore size). Carbohydrate and protein concentrations were determined as previously described^18^. Crude fiber and fat contents were determined using a FiberCap 2021/2023 system (Foss, Denmark) and a ST255 Soxtec system (Foss, UK), respectively. The concentrations of trace metals (Al, Co, Cr, Cu, Fe, Mn, Mo, Ni, W, and Zn) were determined by inductively coupled plasma-optical emission spectrometry (700-ES, Varian, USA). Organic C, H, O, N, and S contents were determined using a FLASH 2000 CHNS/O analyzer (Thermo Scientific, The Netherlands). Excitation-emission matrix (EEM) fluorescence spectroscopy was performed to characterize the residual organic matter in the effluent using a Cary Eclipse spectrofluorometer (Varian, USA) with an excitation wavelength range of 220–500 nm at a scanning interval of 10 nm^19^. All samples from each of the duplicate reactors were analyzed in triplicate, except for EEM analysis.

### DNA preparation and sequencing

Total community DNA for high-throughput sequencing (HTS) analysis was extracted using an ExiProgen automated nucleic acid extractor (Bioneer, Korea) as described previously^3^. The DNA extracted from each sample (200-μL aliquot of mixed liquor suspension after repeated washing by pelleting, supernatant removal, and resuspension in distilled water) was eluted in 100 μL of elution buffer. Archaeal and bacterial 16S rRNA genes were amplified from the purified DNA by polymerase chain reaction (PCR) using 787F/1059R and 338F/805R primer pairs specific for archaea and bacteria, respectively^20^. Each primer was 5′-end labeled with an Illumina adapter sequence. The PCR was conducted under the following thermal cycling conditions: an initial denaturation at 94℃ for 10 min, 30 amplification cycles (94℃ for 30 s, 55℃ for 30 s, and 72℃ for 30 s), and a final extension for 7 min at 72℃. The resulting PCR fragments were purified using AMPure XP beads (Beckman Coulter, USA) to remove non-specific products and impurities and then indexed for library preparation using the Nextera XT Index Kit v2 (Illumina, USA). Each library was checked for DNA concentration using a Qubit dsDNA BR assay kit (Thermofisher, USA), and the archaeal and bacterial libraries were pooled in equimolar concentrations following the Illumina Metagenomic Sequencing Library Preparation instructions (Illumina, USA). HTS of the pooled library mixtures were performed by Macrogen Inc. (Korea) on the Illumina MiSeq platform. After removing raw reads with low quality scores or ambiguous bases, the qualified reads were aligned and clustered into amplicon sequence variants (ASVs) using DADA2 version 1.18.0^21^. The identified ASVs were taxonomically classified against the RDP database using the UCLUST algorithm^22^ in QIIME version 1.9.0^23^. The DNA sequence data reported in this study were deposited in the NCBI Sequence Read Archive under BioProject accession number PRJNA894847. The prediction of functional gene abundances in microbial communities from the 16S rRNA gene sequence data was performed using PICRUSt2 version 2.5.0 against the KEGG database^24^.

### Statistical analysis

For the duplicate experimental reactors, methane production across the experimental phases were statistically compared based on the steady-state data (with at least 10 data points for each phase) using one-way analysis of variance followed by Tukey’s test. This analysis was carried out using Origin Pro 2023 (OriginLab Co., USA).

Archaeal and bacterial matrices were generated from the HTS data by scoring the relative abundance of individual ASVs in each archaeal or bacterial library. Cluster analysis was carried out for each matrix using the Bray–Curtis distance measure in PAST version 4.06^25^. Cluster dendrograms using the unweighted pair group method with arithmetic means algorithm were constructed from the analysis results.

## Results and discussion

### Reactor performance

**Figure 1:**
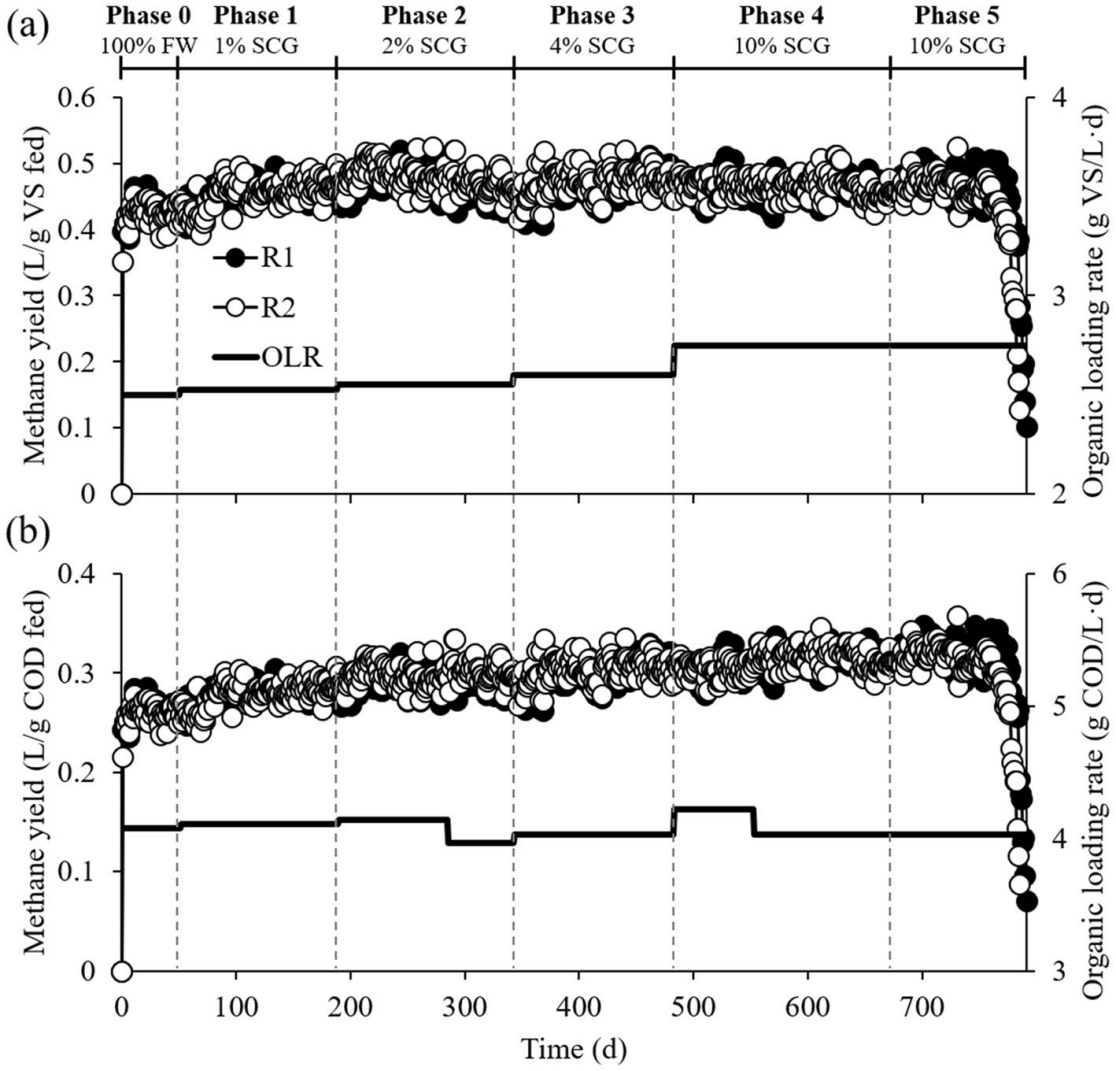
Methane production and organic loading profiles in the duplicate reactors R1 and R2. Methane yield was calculated per unit mass of VS (**a**) and COD (**b**) fed to each reactor. SCG co-feeding ratios are presented as percentages relative to FW on a VS basis.

**Figure 2:**
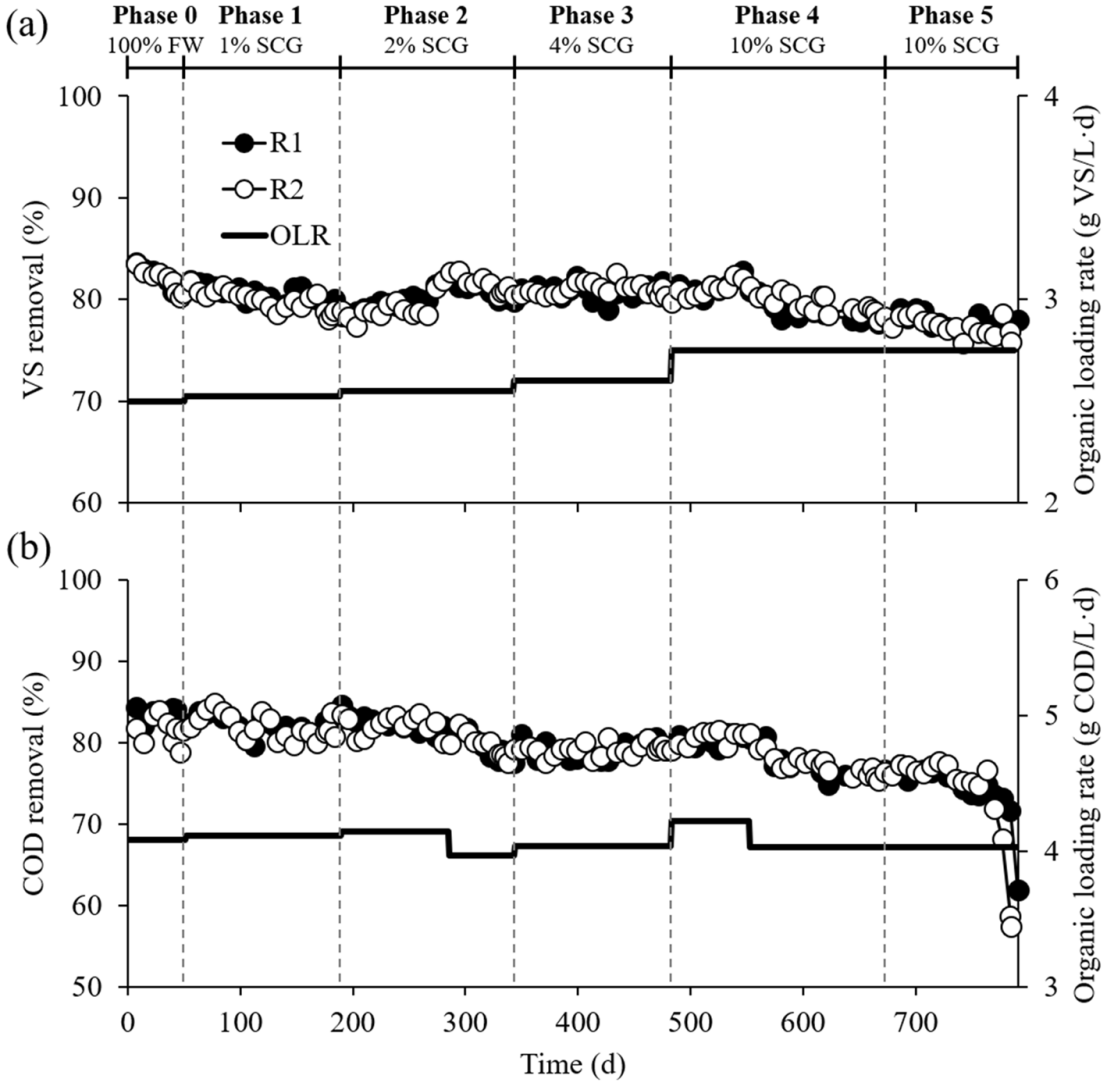
VS (**a**) and COD (**b**) removal profiles in the duplicate reactors R1 and R2. SCG co-feeding ratios are presented as percentages relative to FW on a VS basis.

The duplicate experimental reactors, R1 and R2, were operated for more than 26 months at increasing ratios of SCG to FW in the reactor feed, from 0 to 10% on a VS basis, and the OLR rose from 2.50 to 2.75 g VS/L·d accordingly (see Supplementary Table S2). The reactors were successfully started up and stabilized during Phase 0 fed with FW only, and they performed very similarly to each other throughout the experiment (Figs. 1 and 2). This result indicates the sound replication of the reactor experiments. The steady-state methane yield and organic removal obtained in Phase 0 (Table 1) were comparable to those reported for FW mono-digestion in other studies^3,26^.

**Table 1:** Steady-state performance data in each experimental phase.

	Phase 0		Phase 1		Phase 2		Phase 3		Phase 4	
	R1	R2	R1	R2	R1	R2	R1	R2	R1	R2
Methane yield (L/g VS fed)	0.43 (0.0)^a^	0.43 (0.0)	0.45 (0.0)	0.46 (0.0)	0.46 (0.0)	0.47 (0.0)	0.48 (0.0)	0.47 (0.0)	0.46 (0.0)	0.45 (0.0)
Methane yield (L/g COD fed)	0.26 (0.0)	0.26 (0.0)	0.28 (0.0)	0.28 (0.0)	0.29 (0.0)	0.30 (0.0)	0.31 (0.0)	0.30 (0.0)	0.31 (0.0)	0.31 (0.0)
Methane content (%)	56.5 (0.4)	56.3 (0.3)	56.5 (0.2)	56.7 (0.1)	55.3 (1.0)	55.8 (1.0)	56.1 (0.1)	55.7 (0.2)	56.2 (0.4)	55.8 (0.1)
TS (g/L)	26.1 (0.6)	26.3 (0.8)	28.1 (1.2)	29.3 (1.0)	27.9 (0.5)	27.2 (1.1)	26.3 (0.7)	27.1 (0.8)	30.6 (0.6)	30.6 (0.8)
VS (g/L)	18.8 (0.6)	19.1 (0.7)	20.5 (0.9)	21.6 (0.8)	21.3 (0.5)	20.5 (1.0)	20.4 (0.7)	20.9 (0.7)	24.4 (0.6)	24.0 (0.6)
VS removal (%)	81.1 (0.2)	80.8 (0.7)	79.7 (0.4)	78.5 (0.4)	80.1 (0.1)	80.8 (0.4)	80.5 (0.5)	79.9 (0.5)	77.9 (0.2)	78.2 (0.4)
Total COD (g/L)	27.6 (2.0)	29.7 (2.1)	28.8 (0.3)	29.9 (2.2)	34.5 (0.9)	34.5 (0.8)	32.7 (1.0)	33.3 (0.5)	38.6 (1.0)	38.6 (0.9)
COD removal (%)	82.0 (1.3)	80.6 (1.4)	82.5 (0.2)	81.8 (1.3)	78.2 (0.6)	78.3 (0.5)	79.7 (0.6)	79.4 (0.3)	76.2 (0.3)	76.0 (0.6)
Carbohydrate (g/L)	2.6 (0.1)	2.6 (0.1)	3.4 (0.2)	3.3 (0.1)	3.3 (0.2)	3.2 (0.2)	4.0 (0.1)	4.3 (0.1)	4.0 (0.1)	4.6 (0.3)
Protein (g/L)	17.6 (1.3)	15.3 (2.4)	15.3 (2.7)	15.8 (1.3)	13.9 (0.8)	13.2 (1.5)	14.2 (1.3)	16.0 (0.4)	9.6 (1.3)	9.0 (0.2)
Crude fat (g/L)	n.d.^b^	n.d	0.3 (0.0)	0.4 (0.0)	0.5 (0.0)	0.5 (0.1)	0.6 (0.1)	1.1 (0.1)	0.3 (0.0)	0.2 (0.0)

*TS*, total solids; *VS*, volatile solids; *COD*, chemical oxygen demand.

^a^Standard deviations are in parentheses.

^b^Not determined.

**Figure 3:**
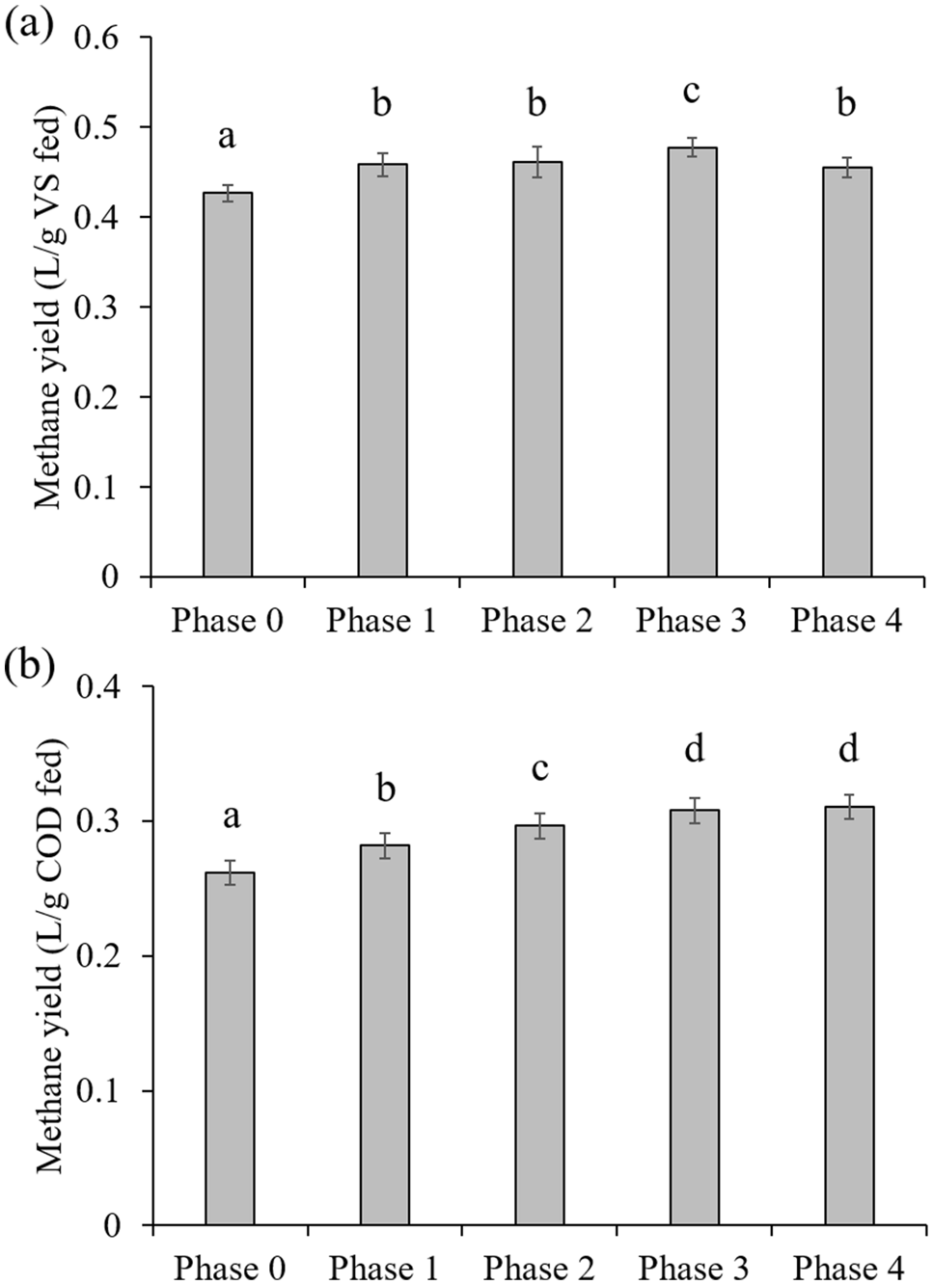
Steady-state methane yields per unit mass of VS (**a**) and COD (**b**) fed in each experimental phase. Data presented are averages of the duplicate reactors R1 and R2. Different letters above bars indicate statistically significant differences (*p* < 0.05, Tukey’s test).

Interestingly, methane yield increased with increasing SCG in the co-feeding ratio range of 1–4% of FW on a VS basis (Phases 1–3) based on both VS and COD fed to the reactors, and no further increase was observed after increasing SCG content to 10% (Phase 4) (Fig. 3). The methane yields obtained in Phases 1–4 were significantly higher than that in Phase 0 (Tukey’s test, *p* < 0.05). This result suggests that co-feeding small amounts of SCG (≤ 10% of FW) was not detrimental but rather beneficial to methanogenic performance under the experimental conditions. Correspondingly, the concentration of residual VFAs remained at very low levels (< 200 mg COD/L) with neutral pH throughout Phases 0–4 (see Supplementary Fig. S1). One point to note is that FW was collected on two occasions due to the long experimental period, and the reactors were fed with the first batch of FW until day 284 and afterwards with the second batch (see Supplementary Table S1). The first and second batches of FW had very similar TS and VS contents but significantly different COD concentrations, implying that the chemical composition and energy content of FW varied. The total COD-to-VS ratio decreased from 1.62 for the first batch of FW to 1.56 for the second, which explains why the methane yield per VS fed (Y_MVSf_) did not increase significantly between Phases 1 and 2, while the methane yield per COD fed (Y_MCODf_) did (Fig. 3). Both Y_MVSf_ and Y_MCODf_ showed an increasing trend with increasing the SCG fraction in the feed over Phases 0–3, despite the decrease in the COD-to-VS ratio of the FW, suggesting that the most efficient co-digestion of SCG and FW can be achieved by co-feeding SCG at 4% of FW (VS basis).

Both reactors failed with significant performance degradation in three turnovers of the HRT during Phase 5, which was conducted under the same conditions as Phase 4 but without supplementing the feed with trace elements (Fe, Ni, and Co; see Subsection "Reactor setup and operation") (Figs. 1 and 2). Accordingly, a serious process imbalance occurred with a sudden accumulation of VFAs, primarily acetate and propionate, accompanied by reactor acidification, during Phase 5 (see Supplementary Fig. S1). These results agree with a previous study by the authors’ group reporting that FW digesters failed after co-feeding SCG with FW at a ratio of 1:10 (VS basis) due to a suspected lack of trace elements^3^. Given that the experimental reactors maintained stable operation for 188 days (nearly five turnovers of the HRT) during Phase 4 with higher methanogenic performance as compared to Phase 0, it is clear that providing additional trace elements is important for the stable co-digestion of SCG and FW, especially in a 1:10 mixture. The deficiency of trace elements in SCG-FW mixtures may be ascribed to the chelation of metal cations by anionic polymeric compounds present in SCG, such as polyphenols and especially melanoidins. Coffee melanoidins effectively chelate metals, especially iron, at low concentrations, and metal ions captured at the melanoidin core become unavailable for microorganisms^27^.

**Figure 4:**
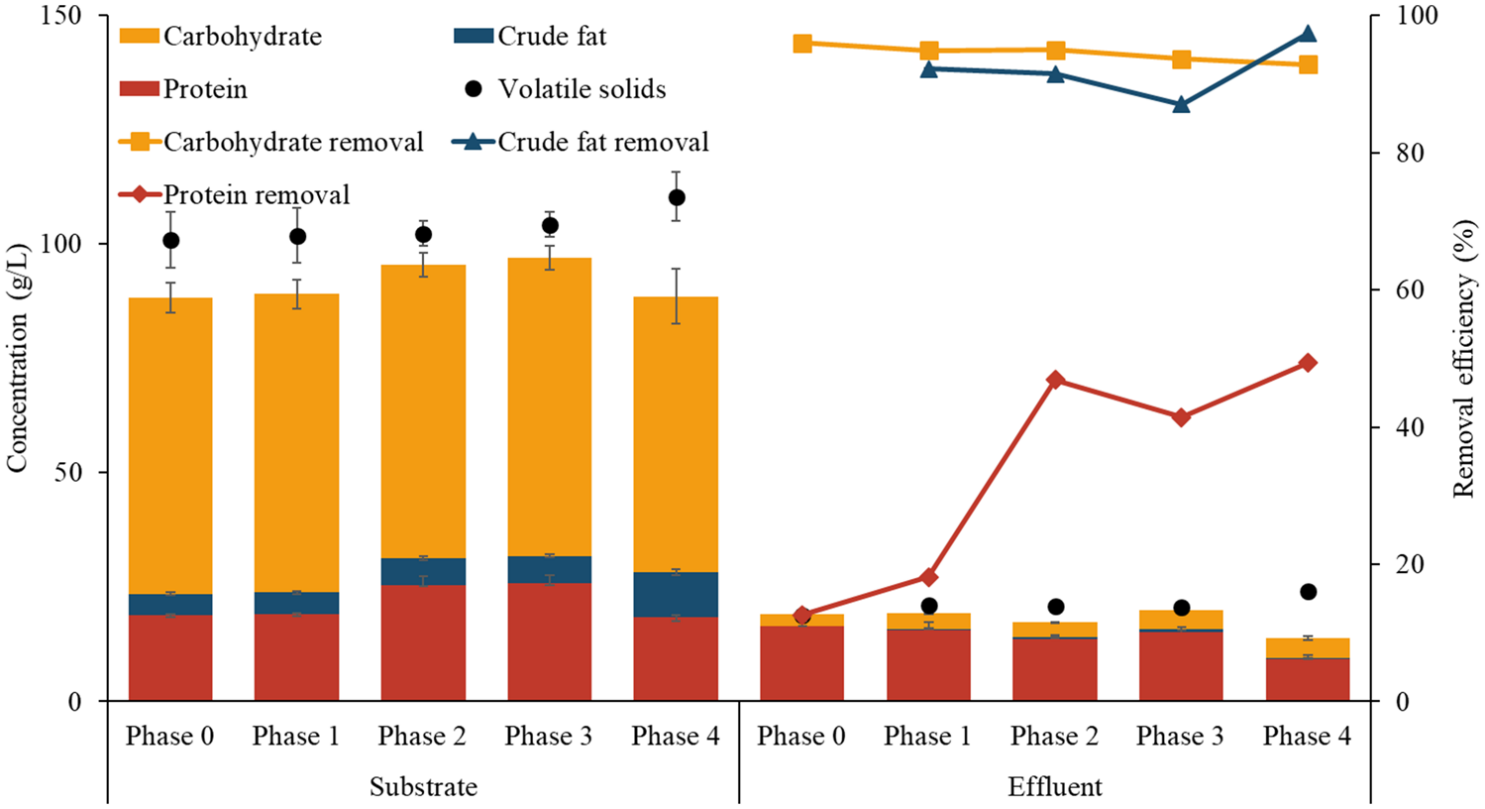
Carbohydrate, crude fat, and protein removal efficiencies in each experimental phase.

It is notable that the removal efficiency of protein increased significantly with the addition of SCG from less than 20% to approximately 50%, while those of carbohydrate and crude fat remained relatively stable between about 90 and 100% (Fig. 4). The markedly higher residual concentration of protein as compared to carbohydrate and fat reflects the fact that protein is the most abundant cellular component of bacteria, accounting for approximately 50–80% of dry weight^28^, and agrees with previous FW-AD studies reporting the significantly lower removal of protein as compared to carbohydrate and fat^29,30^. Although the underlying mechanism is unclear, the enhanced protein degradation appears to have contributed to the increase in methane yield in Phases 1–4 with SCG addition as compared to Phase 0 without it, which deserves further research. Accordingly, protein removal efficiency had a significant positive correlation (Pearson, *p* < 0.05) with the SCG fraction in the feed (*r* = 0.71) as well as methane yield (*r* = 0.90). Further, EEM fluorescence spectroscopy analysis revealed that the abundance of dissolved organic matter, especially protein-like substances, in the effluent decreased greatly across the experimental phases (see Supplementary Fig. S2).

### Microbial community analysis

**Figure 5:**
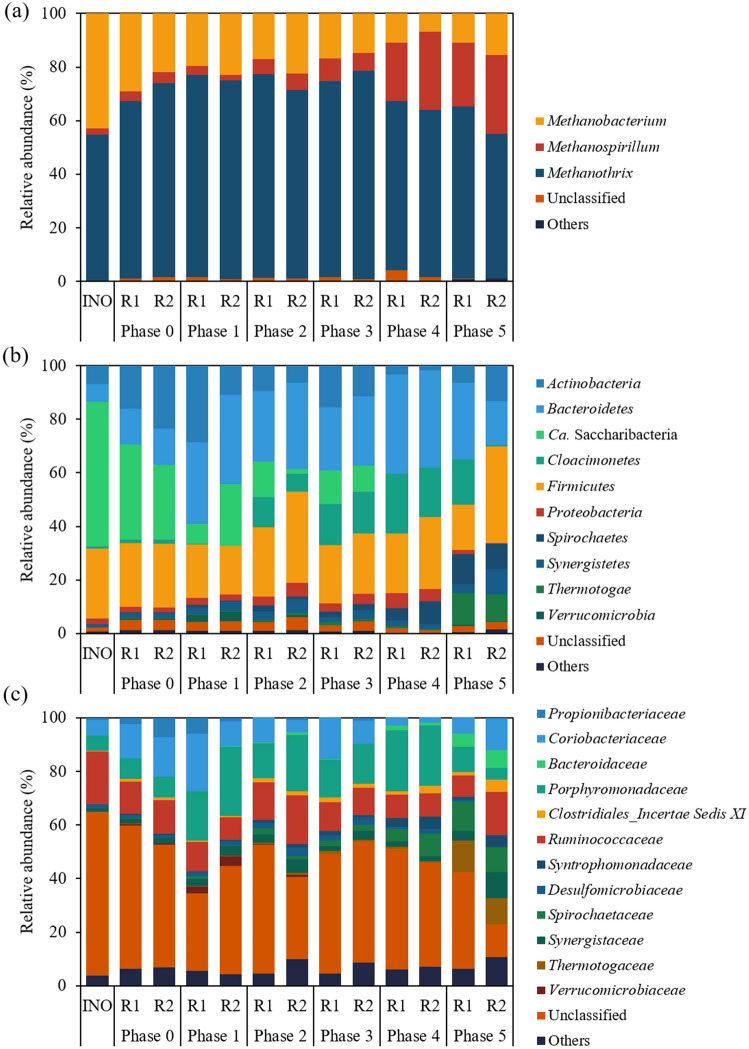
Taxonomic distribution of retrieved archaeal (**a** genus level) and bacterial (**b** phylum level; **c** family level) 16S rRNA gene sequences. Sequences with relative abundance less than 3% in all samples were classified as “Others”. INO, Inoculum.

The HTS analysis of 16S rRNA genes identified 66 archaeal and 2,270 bacterial ASVs from a total of 3,920,971 reads (150,807 ± 32,454 reads/sample) retrieved from the inoculum sludge and reactor samples. The taxonomic affiliations and relative abundances of major ASVs (≥ 3% of the total reads in at least one bacterial or archaeal library) in each library are presented in Table 2. Nearly all archaeal sequences (> 95% in all archaeal libraries) were assigned to three methanogenic genera: *Methanobacterium*, *Methanospirillum*, and *Methanothrix* (Fig. 5a). In terms of 16S rRNA gene concentration, acetotrophic *Methanothrix* was the most abundant in the reactors throughout the experiment (≥ 54.0%). *Methanothrix* accounted for approximately 55% of the archaeal sequences yielded from the inoculum sludge, with most of the remainder belonging to *Methanobacterium*. The relative abundance of *Methanothrix* increased greatly to 66.4–77.6% during Phases 0–3 with stable reactor operation, while that of *Methanobacterium* decreased accordingly. However, the relative abundance of *Methanothrix* decreased markedly during Phases 4 and 5 (≤ 64.0%) with increasing the SCG co-feeding ratio to 10% of FW (VS basis). These results show that acetotrophic methanogenesis, especially by *Methanothrix*, was the primary route for methane production across the experimental phases but the contribution of hydrogenotrophic methanogenesis increased after adding more SCG (> 4% of FW on a VS basis). This change corresponds to the buildup of residual soluble COD and VFAs along with significant performance degradation over Phases 4 and 5 (see Supplementary Fig. S1), given that hydrogenotrophic methanogens often dominate acetotrophic ones under imbalanced conditions due to their greater resistance to VFA inhibition^31,32^. Interestingly, the relative abundance of *Methanospirillum* increased as the SCG fraction in the feed increased, and it became the dominant hydrogenotrophic methanogen over *Methanobacterium* during Phases 4 and 5. While the exact cause remains uncertain, this dominance shift could be associated with the accumulation of VFAs, as previous studies have observed the thriving of *Methanospirillum* as the major methanogen under VFAs-enriched or organic overloading conditions^33,34^.

**Table 2:**
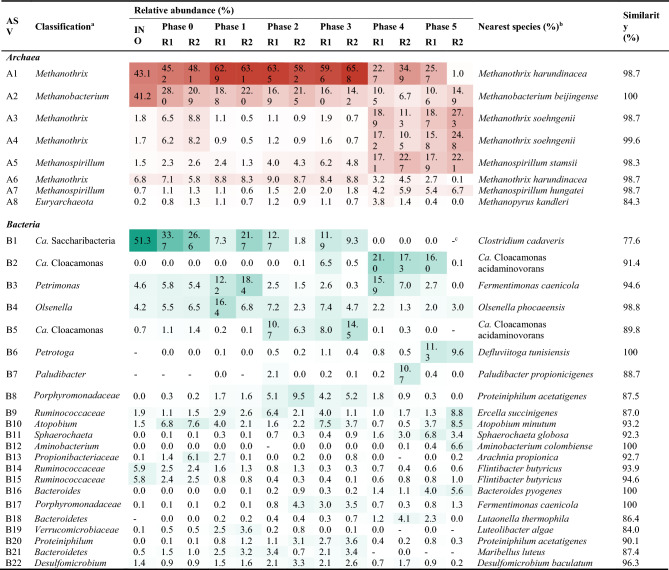
Relative abundance and taxonomic affiliation of major archaeal and bacterial ASVs (> 3% relative abundance at least one library).

Cells with relative abundance values are colored in a heatmap-like fashion (red for archaea; cyan for bacteria). INO, inoculum.

^a^The lowest taxonomic rank assigned against the Ribosomal Database Project database at a confidence threshold of 80%.

^b^Identified by BLAST search against the National Center for Biotechnology Information nucleotide database.

^c^Not detected at all (a zero read). Note that ‘0.0′ means a non-zero read but very low relative abundance below 0.1%.

Most bacterial sequences (> 94% in all bacterial libraries) were assigned to ten major phyla (≥ 3% relative abundance in at least one bacterial library), while 1.0–4.8% of the total bacterial reads were unclassifiable even at the phylum level for each sample. The bacterial communities in the reactors underwent considerable changes in composition with the addition of SCG. The inoculum bacterial community was dominated by ASV B1 (51.3% of the total reads) assigned to *Ca.* Saccharibacteria (Table 2), whose members can hydrolyze various organic compounds in wastewater treatment systems^35,36^. However, its relative abundance decreased over the experimental phases and became less than 0.1% during Phases 4 and 5 in both reactors, which may be related to the considerable reduction in VS and COD removal efficiencies during these phases (Fig. 2).

Meanwhile, the relative abundance of *Ca*. Cloacimonetes (ASVs B2 and B5) and *Bacteroidetes* (ASVs B3, B7, B8, B16, B17, B18, B20, and B21) increased after co-feeding SCG (Fig. 5b). Members of these phyla can ferment amino acids, sugars, and alcohols into VFAs^37^. Both ASVs B2 and B5 were assigned to the genus *Ca*. Cloacamonas including H_2_-producing syntrophs oxidizing propionate to acetate and CO_2_, and members of this genus have been reported to participate in the degradation of cellulosic materials during AD^38–40^. Therefore, these *Ca*. Cloacamonas-related bacteria likely contributed to the degradation of SCG fibers, corresponding to the increase in their relative abundance after co-feeding SCG (Table 2 and Fig. 5b). The increment of *Ca*. Cloacamonas, and another hydrogenic genus *Petrotoga* (ASV B6) producing H_2_ from sugar fermentation^41^, over Phases 2–5 could be related to the increased proportion of hydrogenotrophic methanogens in the later phases fed with greater amounts of SCG (Fig. 5a). The dominance shifts between H_2_-producing populations, for example, from ASVs B5 to B2 between Phases 3 and 4, seem to reflect the differences in their tolerance and response to the accumulation of VFAs and H_2_ (see Supplementary Fig. S1). The relative abundance of *Porphyromonadaceae* (ASVs B3, B8, B17, and B20) increased with the addition of SCG over Phases 1–4. Members of this family can ferment carbohydrates and proteins into various organic acids^42^. The increased relative abundance of the aforementioned bacteria likely resulted from the addition of SCG (Fig. 5c).

ASV B4 was assigned to the genus *Olsenella*, known to degrade carbohydrates and produce lactic acid^43^. ASVs B9 and B10 were affiliated with the family *Ruminococcaceae* and the genus *Atopobium*, respectively, whose members are cellulolytic and commonly observed in animal guts^3,16^. Therefore, the bacterial populations represented by these three ASVs were potentially involved in the decomposition of SCG, although they exhibited no distinct trend in their relative abundance following the addition of SCG. Additionally, the emergence of *Ruminococcaceae*, *Atopobium*, *Paludibacter*, and *Petrimonas* under SCG feeding conditions was consistent with observations from previous studies by the authors’ group^3,42^, although their specific roles remain unclear.

### Changes in microbial community structure and function

Clustering analysis based on the distribution of individual ASVs in the sequenced libraries revealed that co-feeding SCG in small amounts (1–10% of FW on a VS basis) had a significant influence on the evolution of the microbial community structure in the reactors. Both archaeal and bacterial cluster dendrograms were clearly divided into two main clusters: one containing the community structures of Phases 0–3 and the other containing those of Phases 4 and 5 (see Supplementary Fig. S3). This result indicates that the increase in the amount of SCG relative to FW from 4 to 10% (VS basis) between Phases 3 and 4 led to significant changes in both the archaeal and bacterial community structures. Structural changes were more pronounced in the bacterial than the archaeal communities in both reactors, which could be partly related to the less dynamic and less diverse nature of archaea as compared to bacteria in methanogenic systems^44^. The bacterial community maintained significantly higher diversity (Shannon index (*H'*) = 4.0–5.1) than the archaeal community (*H'* = 1.2–2.1) throughout the experiment in both reactors (see Supplementary Fig. S4). Interestingly, the diversity of the bacterial community was greater after the addition of SCG (Phases 1–5) than before (Phase 0 and the inoculum sludge), implying that co-feeding SCG supported the growth of more diverse bacteria. Meanwhile, the archaeal community *H'* decreased between Phases 0 and 1 and remained at the reduced levels during Phases 1–3, which can be attributed to the strong dominance of the community by one population (*Methanothrix*-related ASV A1) with 58.2–65.8% relative abundance during these phases (Table 2). Given the simple archaeal community composition, the increase in archaeal diversity during Phases 4 and 5 appears to reflect the increment of other archaeal populations than ASV A1, especially *Methanospirillum*-related ASVs (i.e., a less uneven distribution).

**Figure 6:**
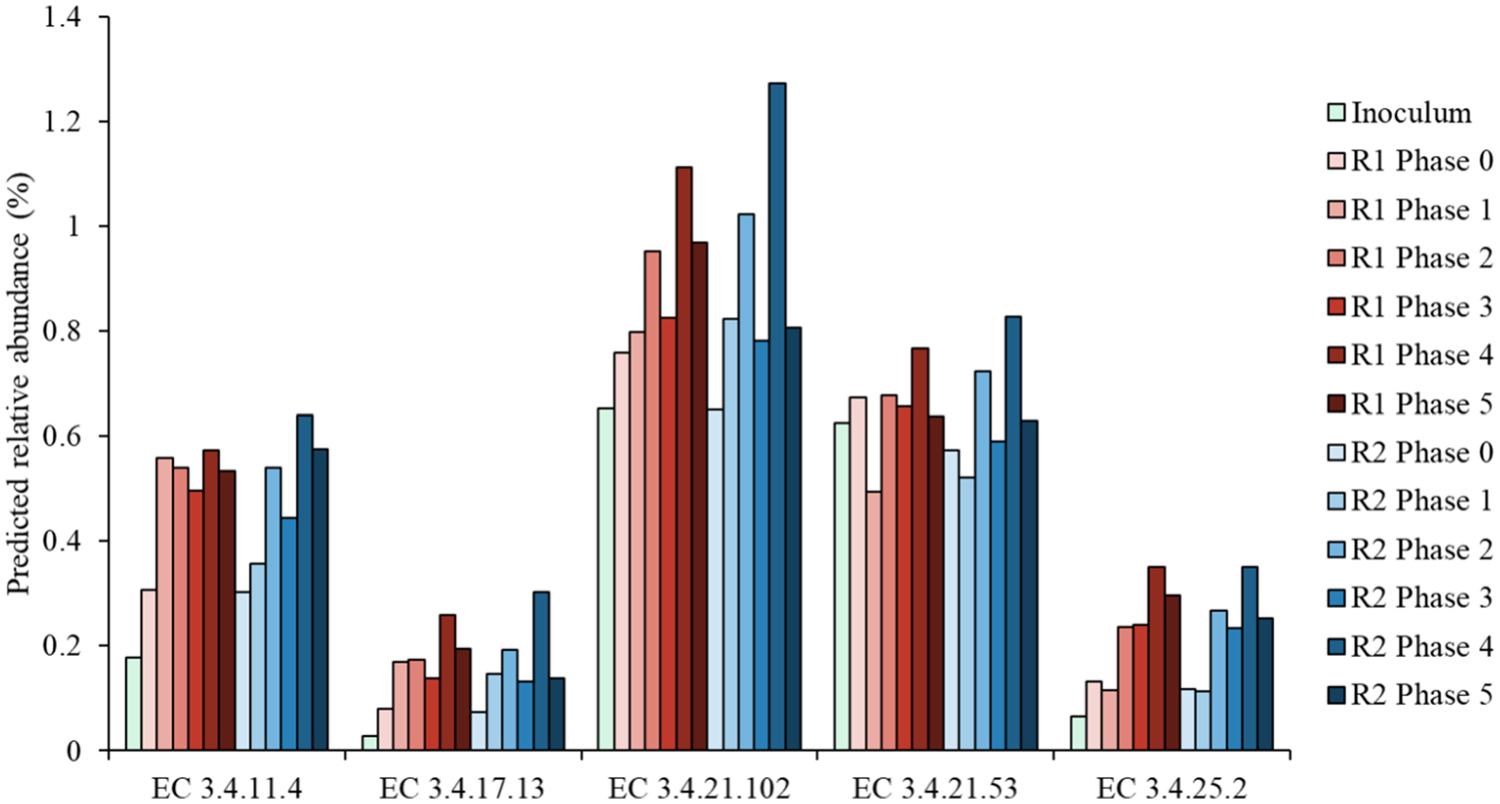
Predicted protease genes (indicated by EC numbers) potentially related to the enhancement of protein degradation under SCG co-feeding conditions.

Functional potential analysis by PICRUSt2 was performed to understand the enhancement of protein removal efficiency, leading to increased methane production, with co-feeding SCG at the functional gene level. The total predicted abundance of protease genes (i.e., the sum of all protease genes) did not correlate significantly with protein removal efficiency (Pearson, *p* = 0.27). The predicted protease genes were individually tested to identify the genes putatively involved in the enhanced degradation of protein, and five of them showed significant positive correlations with both protein removal efficiency and the addition of SCG (Pearson, *p* < 0.05) (Fig. 6). Three and two of the five identified genes encode endopeptidases (endopeptidase La [EC 3.4.21.53], C-terminal processing protease [EC 3.4.21.102], and HslU–HslV peptidase [EC 3.4.25.2]) and exopeptidases (muramoyltetrapeptide carboxypeptidase [EC 3.4.17.13] and tripeptide aminopeptidase [EC 3.4.11.4]), respectively, suggesting that the enhanced protein degradation observed under SCG co-feeding conditions may be related to facilitated peptide hydrolysis. Notably, 30 of 2,270 bacterial ASVs were identified as having a significant positive correlation with both protein removal efficiency and the amount of SCG added, as well as carrying one or more of these predicted genes. Half of the ASVs belonged to two families *Ruminococcaceae* (10) and *Syntrophomonadaceae* (5) of the fermentative order *Clostridiales*, and six belonged to the family *Spirochaetaceae*. Six of the remainder were assigned to the families *Alcaligenaceae* (1), *Bacteroidaceae* (1), *Porphyromonadaceae* (2), *Synergistaceae* (1), and *Thermotogaceae* (1), and the other three were classifiable only at the phylum level as *Bacteroidetes*. Members of the families mentioned above commonly occur in methanogenic systems and involved in the fermentative degradation of organic matter, including protein, to VFAs and/or H_2_/CO_2_^13,45–47^. Furthermore, the co-occurrence analysis (Pearson, *p* < 0.05) of major ASVs (Table 2) identified ASV B16, which was among the three major ASVs predicted to possess the protease genes of interest (B6, B11, and B16), as the most influential node with 16 edges (nine positive and seven negative interactions) with other ASVs. In fact, ASV B16 was assigned to the genus *Bacteroides*, belonging to the family *Bacteroidaceae*, and showed 100% sequence identity with a saccharolytic and proteolytic species *Bacteroides pyogenes*^48^. These results suggest that the proteolytic bacteria discussed above may have contributed to the enhancement of protein removal efficiency with the addition of SCG in the experimental reactors.

## Conclusions

Co-digesting small amounts of SCG with FW (1:100–1:10 on a VS basis) was examined to explore the possibility of treating SCG using the spare capacity of existing anaerobic digesters. Methane yield increased with the increasing addition of SCG up to 4% of FW, while co-feeding more SCG caused no further improvement. Process failure occurred when SCG was co-fed at 10% of FW without trace element supplementation. The enhanced methanation seems to reflect the increased protein removal (peptide hydrolysis) under SCG co-feeding conditions. Co-feeding appropriate small amounts of SCG to FW digesters can be an option for sustainable SCG management.

### Supplementary Information

Supplementary Tables.

Supplementary Information 1.

## Data Availability

The datasets used and analyzed during the current study available from the corresponding author on reasonable requests.

## References

[1] Bio-bean. *Zero Waste Week: There’s no such thing as waste coffee!, Bio-bean.*, https://www.bio-bean.com/news-post/zero-waste-week-put-your-waste-coffee-grounds-to-work/ (2022).

[2] National Assembly Research Service of Korea. *커피찌꺼기 수거체계 확립을 통한 바이오에너지 연료자원화 방안* https://www.nars.go.kr/report/view.do?cmsCode=CM0043&brdSeq=31496 (2020).

[3] Kim D, Kim H, Kim J, Lee C (2019). Co-feeding spent coffee grounds in anaerobic food waste digesters: Effects of co-substrate and stabilization strategy. Bioresour. Technol..

[4] Kim D, Choi H, Lee C (2022). Pretreatment of spent coffee grounds with alkaline soju bottle-washing wastewater for enhanced biomethanation. Biomass Convers. Biorefin..

[5] Fernandez N, Forster CF (1993). A study of the operation of mesophilic and thermophilic anaerobic filters treating a synthetic coffee waste. Bioresour. Technol..

[6] Dinsdale RM, Hawkes FR, Hawkes DL (1996). The mesophilic and thermophilic anaerobic digestion of coffee waste containing coffee grounds. Water Res..

[7] Kim J, Kim H, Baek G, Lee C (2017). Anaerobic co-digestion of spent coffee grounds with different waste feedstocks for biogas production. Waste Manage..

[8] Atelge MR (2021). Anaerobic co-digestion of oil-extracted spent coffee grounds with various wastes: Experimental and kinetic modeling studies. Bioresour. Technol..

[9] Ike M (2010). Microbial population dynamics during startup of a full-scale anaerobic digester treating industrial food waste in Kyoto eco-energy project. Bioresour. Technol..

[10] Li Q (2015). Kinetic characterization of thermophilic and mesophilic anaerobic digestion for coffee grounds and waste activated sludge. Waste Manage..

[11] Qiao W, Mohammad S, Takayanagi K, Li YYJRA (2015). Thermophilic anaerobic co-digestion of coffee grounds and excess sludge: long term process stability and energy production. RSC Adv..

[12] Kim J, Kim H, Lee C (2017). *Ulva* biomass as a co-substrate for stable anaerobic digestion of spent coffee grounds in continuous mode. Bioresour. Technol..

[13] Zhang W, Wang X, Xing W, Li R, Yang T (2021). Responses of anaerobic digestion of food waste to coupling effects of inoculum origins, organic loads and pH control under high load: Process performance and microbial characteristics. J. Environ. Manage..

[14] Korea Ministry of Environment. *전국폐기물 발생 및 처리현황*, https://www.recycling-info.or.kr/rrs/stat/envStatDetail.do?menuNo=M13020201&pageIndex=1&bbsId=BBSMSTR_000000000002&s_nttSj=KEC005&nttId=1200&searchBgnDe=&searchEndDe= (2022).

[15] Prabhudessai V, Ganguly A, Mutnuri S (2009). Effect of caffeine and saponin on anaerobic digestion of food waste. Ann. Microbiol..

[16] Jo Y (2021). Long-term effectiveness of bioaugmentation with rumen culture in continuous anaerobic digestion of food and vegetable wastes under feed composition fluctuations. Bioresour. Technol..

[17] APHA-AWWA-WEF. *Standard methods for the examination of water and wastewater*. 21st ed. edn, (American Public Health Association, 2005).

[18] Baek G, Kim D, Kim J, Kim H, Lee C (2020). Treatment of cattle manure by anaerobic co-digestion with food waste and pig manure: Methane yield and synergistic effect. Int. J. Environ. Res. Public Health.

[19] Park S (2019). Evaluating membrane fouling potentials of dissolved organic matter in brackish water. Water Res..

[20] Yu Y, Lee C, Kim J, Hwang S (2005). Group-specific primer and probe sets to detect methanogenic communities using quantitative real-time polymerase chain reaction. Biotechnol. Bioeng..

[21] Callahan BJ (2016). DADA2: High-resolution sample inference from Illumina amplicon data. Nat. Methods.

[22] Edgar RC (2010). Search and clustering orders of magnitude faster than BLAST. Bioinform..

[23] Caporaso JG (2010). QIIME allows analysis of high-throughput community sequencing data. Nat. Methods.

[24] Douglas GM (2020). PICRUSt2 for prediction of metagenome functions. Nat. Biotechnol..

[25] Hammer Ø, Harper DA, Ryan PDJPE (2001). PAST: Paleontological statistics software package for education and data analysis. Palaeontol. Electronica.

[26] Pramanik SK, Suja FB, Zain SM, Pramanik BK (2019). The anaerobic digestion process of biogas production from food waste: Prospects and constraints. Bioresour. Technol. Rep..

[27] Rufián-Henares JA, de la Cueva SP (2009). Antimicrobial activity of coffee melanoidins—A study of their metal-chelating properties. J. Agric. Food Chem..

[28] Ritala A, Häkkinen ST, Toivari M, Wiebe MG (2017). Single cell protein—State-of-the-art, industrial landscape and patents 2001–2016. Front. Microbiol..

[29] Qin Y, Wu J, Xiao B, Hojo T, Li YY (2018). Biogas recovery from two-phase anaerobic digestion of food waste and paper waste: Optimization of paper waste addition. Sci. Total Environ..

[30] Qi WK (2021). Detailed composition evolution of food waste in an intermittent self-agitation anaerobic digestion baffled reactor. Bioresour. Technol..

[31] Xu Z (2014). *In situ* volatile fatty acids influence biogas generation from kitchen wastes by anaerobic digestion. Bioresour. Technol..

[32] Vanwonterghem I, Jensen PD, Rabaey K, Tyson GW (2015). Temperature and solids retention time control microbial population dynamics and volatile fatty acid production in replicated anaerobic digesters. Sci. Rep..

[33] Cao L, Cox CD, He Q (2021). Patterns of syntrophic interactions in methanogenic conversion of propionate. Appl. Microbiol. Biotechnol..

[34] Lerm S (2012). Archaeal community composition affects the function of anaerobic co-digesters in response to organic overload. Waste Manage..

[35] Lin X (2020). Influence of polyether sulfone microplastics and bisphenol A on anaerobic granular sludge: Performance evaluation and microbial community characterization. Ecotoxic. Environ. Saf..

[36] Wen Q, Liu B, Li F, Chen Z (2020). Substrate strategy optimization for polyhydroxyalkanoates producing culture enrichment from crude glycerol. Bioresour. Technol..

[37] Chen L (2021). Effect of the organic loading rates increase on process stability and microbial community composition during the anaerobic digestion of fresh vinegar residue. Waste Biomass Valorization.

[38] Pelletier E (2008). *Candidatus* Cloacamonas Acidaminovorans: Genome sequence reconstruction provides a first glimpse of a new bacterial division. J. Bacteriol..

[39] Weimann A (2016). From genomes to phenotypes: Traitar, the microbial trait analyzer. mSystems.

[40] Cheng J (2020). Nanoscale zero-valent iron improved lactic acid degradation to produce methane through anaerobic digestion. Bioresour. Technol..

[41] Ghanimeh SA, Al-Sanioura DN, Saikaly PE, El-Fadel M (2018). Correlation between system performance and bacterial composition under varied mixing intensity in thermophilic anaerobic digestion of food waste. J. Environ. Manage..

[42] Kim D, Kim J, Lee C (2018). Effect of mild-temperature thermo-alkaline pretreatment on the solubilization and anaerobic digestion of spent coffee grounds. Energies.

[43] Li BY (2022). Production of volatile fatty acid from fruit waste by anaerobic digestion at high organic loading rates: Performance and microbial community characteristics. Bioresour. Technol..

[44] Choi G, Kim J, Lee S, Lee C (2018). Anaerobic co-digestion of high-strength organic wastes pretreated by thermal hydrolysis. Bioresour. Technol..

[45] Palatsi J, Viñas M, Guivernau M, Fernandez B, Flotats X (2011). Anaerobic digestion of slaughterhouse waste: Main process limitations and microbial community interactions. Bioresour. Technol..

[46] Solli L, Håvelsrud OE, Horn SJ, Rike AG (2014). A metagenomic study of the microbial communities in four parallel biogas reactors. Biotechnol. Biofuels.

[47] Niu Q, Takemura Y, Kubota K, Li YY (2015). Comparing mesophilic and thermophilic anaerobic digestion of chicken manure: Microbial community dynamics and process resilience. Waste Manage..

[48] Ziganshina EE, Belostotskiy DE, Bulynina SS, Ziganshin AM (2021). Effect of magnetite on anaerobic digestion of distillers grains and beet pulp: Operation of reactors and microbial community dynamics. J. Biosci. Bioeng..

