# Supplementary material for: Enhanced methane production with co-feeding spent coffee grounds using spare capacity of existing anaerobic food waste digesters
Source: Sci Rep. 2024 Feb 23;14:4472. doi: 10.1038/s41598-024-54610-y (PMC10891051; doi:10.1038/s41598-024-54610-y)
Supplement: Supplementary file 1 — Supplementary Tables. [file 41598_2024_54610_MOESM1_ESM.pdf]

Enhanced methane production with co-feeding spent coffee grounds using spare capacity of existing anaerobic food waste digesters

Danbee Kim[a, c], Junho Cha[a], Changsoo Lee[a, b, *]

[a] Department of Urban and Environmental Engineering, Ulsan National Institute of Science and Technology (UNIST), 50 UNIST-gil, Eonyang-eup, Ulju-gun, Ulsan 44919, Republic of Korea

[b] Graduate School of Carbon Neutrality, Ulsan National Institute of Science and Technology (UNIST), 50 UNIST-gil, Eonyang-eup, Ulju-gun, Ulsan 44919, Republic of Korea

[c] Gwangju Clean Energy Research Center, Korea Institute of Energy Research, 25, Samso-ro 270beon-gil, Buk-gu, Gwangju 61003, Republic of Korea

* Corresponding author.

Tel.: +82 52 217 2822; Fax: +82 52 217 2819.

E-mail address: cslee@unist.ac.kr (C. Lee).

Table S1. Physicochemical characteristics of the base substrate and co-substrate used.

	Food waste[a]	Spent coffee grounds[b]
Total solids (TS)	105.3–107.7 g/L	0.96 ± 0.0 g/g
Volatile solids	100.3–100.9 g/L	0.99 ± 0.0 g/g TS
Total chemical oxygen demand	149.1–160.2 g/L	1.20 ± 0.1 g/g
pH	4.4–4.6	6.3[c]
Carbohydrate	55.4–64.8 g/L	0.46 ± 0.2 g/g
Protein	16.9–25.2 g/L	0.13 ± 0.0 g/g
Crude fat	4.6–8.6 g/L	0.13 ± 0.0 g/g
Crude fiber	22.7–33.9% TS	56 ± 0.4% TS
C	46.3–50.7% TS	52.5 ± 0.6% TS
H	6.5–7.1% TS	7.0 ± 0.1% TS
O	30.2–38.0% TS	36.3 ± 0.6% TS
N	2.9–4.7% TS	2.4 ± 0.0% TS
S	Not detected	Not detected

[a] Adjusted to a VS concentration of 100 g/L with distilled water. Given as ranges of mean values from two batches collected at different sampling occasions.

[b] Raw material was dried spent coffee grounds at 55°C for over 48 h.

[c] Measured in a 20 g dry weight/L distilled water slurry.

Table S2. Reactor operating conditions for each experimental phase.

	Phase 0	Phase 1	Phase 2	Phase 3	Phase 4	Phase 5
Period (days)	0–50	51–188	189–342	343–482	483–671	672–791
Substrate composition	FW only	FW + SCG	FW + SCG	FW + SCG	FW + SCG	FW+SCG
SCG co-feeding ratio (% of FW, VS basis)	0	1	2	4	10	10
Organic loading rate (g VS/L·d)	2.5	2.525	2.55	2.6	2.75	2.75
Hydraulic retention time (days)	40	40	40	40	40	40

FW, food waste; SCG, spent coffee grounds.

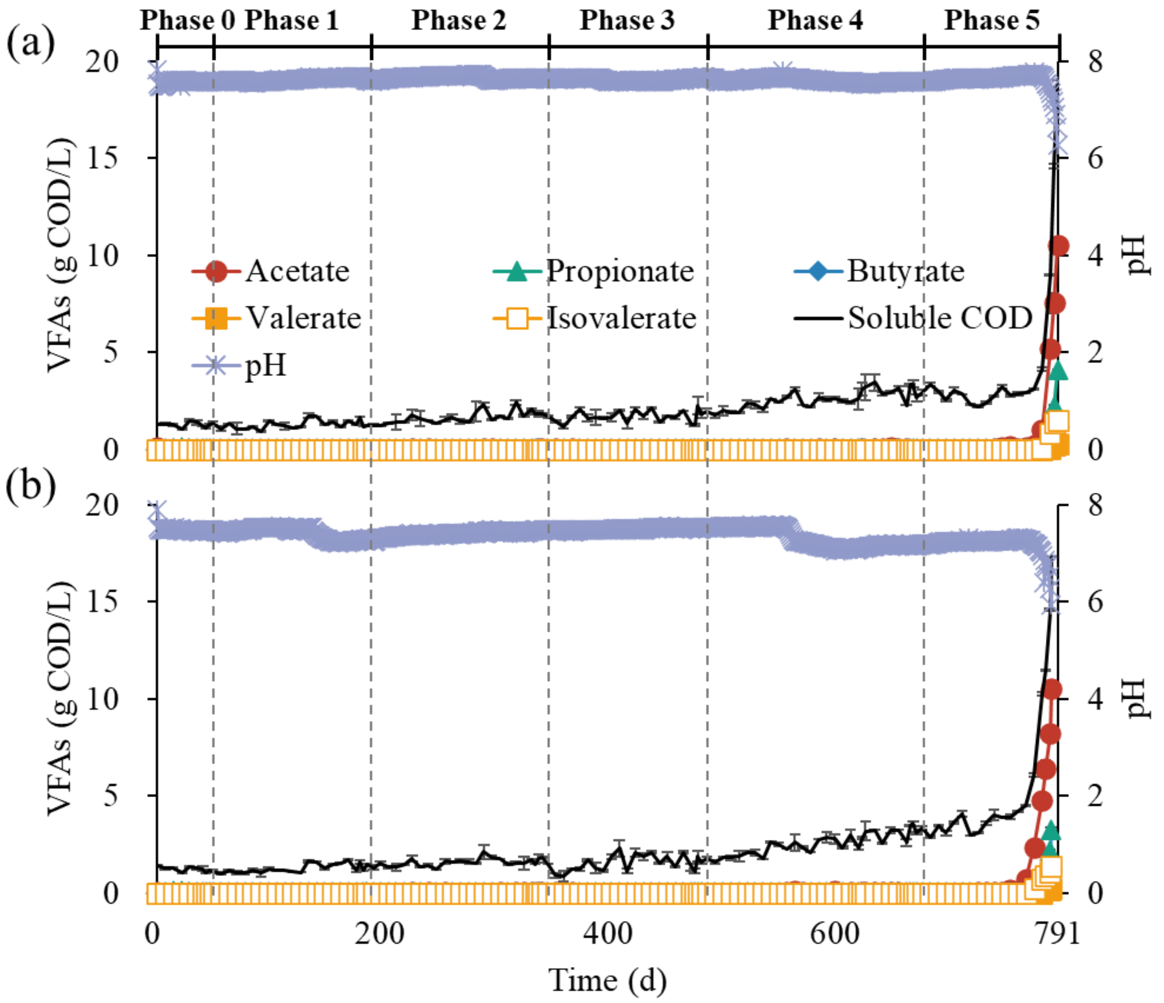

Fig. S1. Changes in residual volatile fatty acids (VFAs) concentrations and pH profile in the duplicate reactors R1 (A) and R2 (B).

Table S3. Concentrations of trace metals in each experimental phase for the duplicate reactors R1 and R2.

Reactor /Phase		Al	Co	Cr	Cu	Fe	Mn	Ni	Zn	Mo	W
R1	0	1.14 (0.07)	0.89 (0.07)	0.01 (0.00)	0.37 (0.03)	47.15 (2.78)	0.64 (0.05)	0.50 (0.05)	2.93 (0.21)	0.03 (0.01)	0.04 (0.03)
	1	1.12 (0.01)	1.30 (0.03)	0.01 (0.00)	0.35 (0.00)	71.50 (0.57)	0.79 (0.01)	0.70 (0.01)	2.01 (0.01)	0.03 (0.01)	Not detected
	2	1.18 (0.03)	1.01 (0.03)	0.02 (0.01)	0.28 (0.01)	59.00 (1.26)	0.69 (0.01)	0.51 (0.02)	2.20 (0.04)	0.02 (0.01)	0.02 (0.02)
	3	1.06 (0.02)	1.45 (0.02)	0.04 (0.00)	0.43 (0.01)	91.00 (0.94)	0.59 (0.01)	1.08 (0.02)	2.76 (0.02)	0.05 (0.01)	0.06 (0.02)
	4	1.48 (0.02)	1.40 (0.02)	0.14 (0.01)	0.54 (0.01)	90.00 (0.81)	0.93 (0.01)	0.86 (0.01)	2.83 (0.04)	0.04 (0.01)	0.03 (0.03)
	5	1.05 (0.01)	0.10 (0.01)	0.01 (0.01)	0.54 (0.01)	8.75 (0.03)	1.45 (0.01)	0.03 (0.03)	3.29 (0.02)	0.11 (0.01)	0.02 (0.03)
R2	0	1.16 (0.03)	0.85 (0.02)	0.01 (0.00)	0.37 (0.00)	45.00 (0.54)	0.65 (0.01)	0.46 (0.02)	2.43 (0.04)	0.03 (0.01)	0.02 (0.03)
	1	1.41 (0.03)	1.53 (0.01)	0.01 (0.01)	0.38 (0.01)	92.00 (1.18)	1.48 (0.02)	0.83 (0.02)	3.75 (0.06)	0.04 (0.01)	0.02 (0.02)
	2	2.31 (0.05)	1.96 (0.04)	0.03 (0.01)	0.49 (0.01)	110.00 (2.87)	0.92 (0.02)	1.04 (0.04)	3.58 (0.08)	0.04 (0.01)	0.04 (0.03)
	3	1.17 (0.06)	1.48 (0.06)	0.04 (0.01)	0.42 (2.90)	92.00 (2.90)	0.76 (0.00)	1.10 (0.04)	2.90(0.13)	0.04 (0.01)	0.03 (0.03)
	4	2.56 (0.04)	1.42 (0.03)	0.11 (0.01)	0.60 (0.01)	94.00 (1.32)	0.97 (0.01)	0.87 (0.01)	3.10 (0.03)	0.04 (0.01)	0.03 (0.02)
	5	1.37 (0.02)	0.11 (0.01)	0.05 (0.01)	0.59 (0.01)	13.50 (0.09)	1.46 (0.01)	0.06 (0.01)	3.58 (0.03)	0.11 (0.01)	0.05 (0.02)

Values are expressed in the unit of mg/L, and standard deviations are in parentheses.

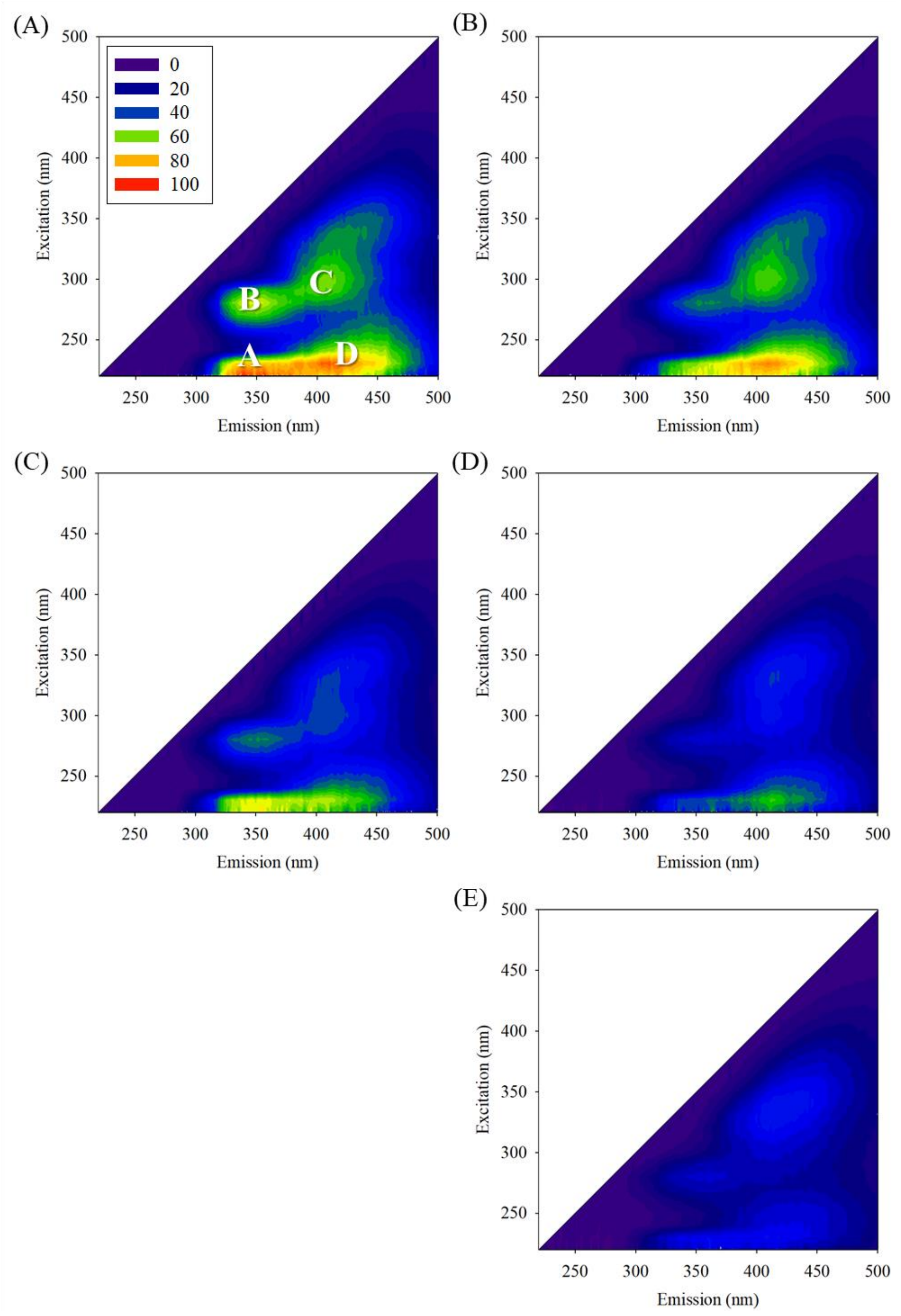

Fig. S2. Excitation-emission matrix fluorescence spectra of R1 effluent samples taken in Phase 0 (A), Phase 1 (B), Phase 2 (C), Phase 3 (D), and Phase 4 (E). Peak A, aromatic protein-like substances; Peak B, microbial byproduct-like substances; Peak C, humic acid-like substances; Peak D, fulvic acid-like substances.

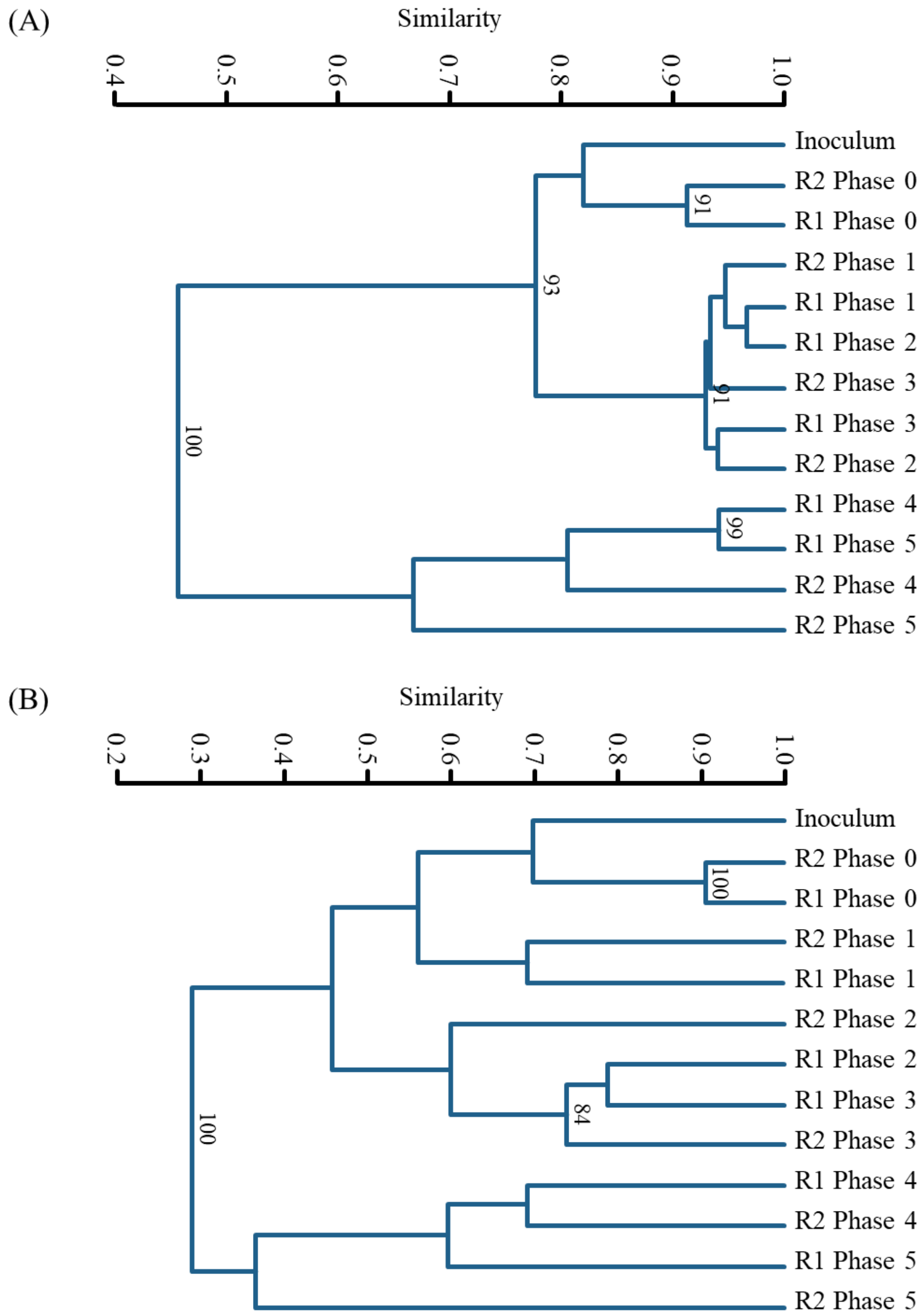

Fig. S3. Cluster dendrograms constructed from the ASV distribution in the archaeal (A) and bacterial (B) 16S rRNA gene libraries. Each library is labeled with the corresponding reactor name and experimental phase. Bootstrap values higher than 80% (1,000 replicates) are shown.

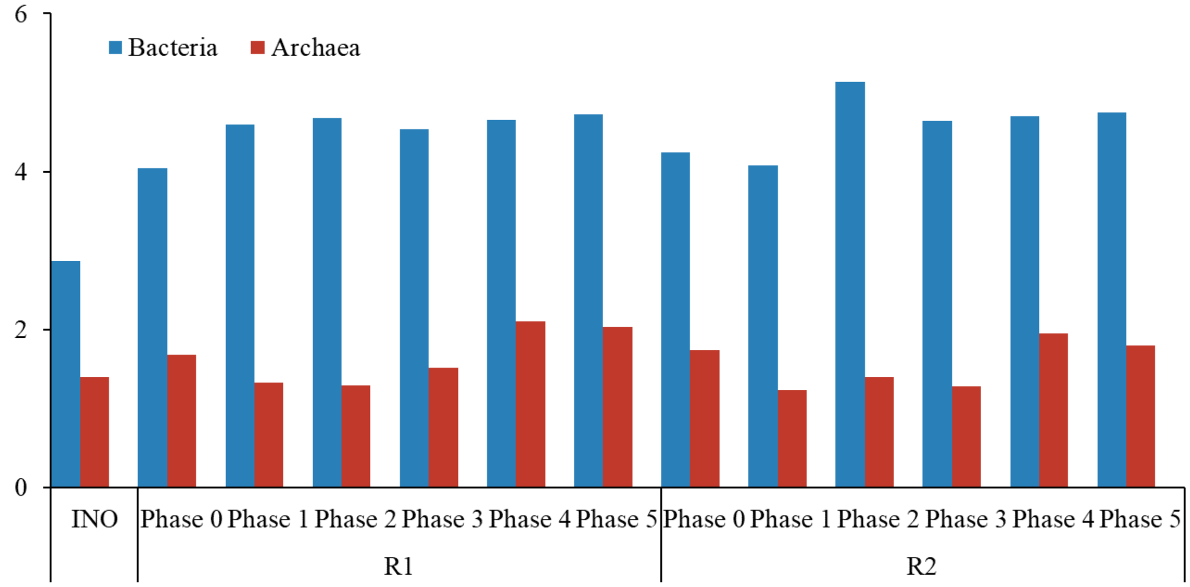

Fig. S4. Shannon's diversity indices of the bacterial and archaeal communities estimated from the ASV profiles.
